# The hidden base of the research pyramid

**DOI:** 10.4103/0972-124X.75907

**Published:** 2010

**Authors:** D. Arunachalam

**Affiliations:** *Editor, Journal of Indian Society of Periodontology, H 11 A South Avenue, Thiruvanmiyur, Chennai - 600 041, India. E-mail: editor@jisponline.com*


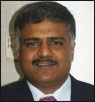


A few days ago, I was an attendee at the 23^rd^ Annual Conference of the Indian Society of Dental Research (ISDR). This year, the meeting at Chennai was hosted by the Chettinad Dental College, and frankly, you would have to see it to believe the behemoth of an institution that it is, combined with the swankiness of technology and facility and the general culture that permeates the institution. It somewhat reminded me of the IITs and the IIMs which still remain the premier educational institutions in India.

It was quite hard to believe that in 23 years of its existence, we had heard so little about an organization like the ISDR, and the conference this year was such a mega turnaround that it stood out and demanded to be noticed. One of our own, Dr. Gururaja Rao, is the President of the ISDR and Dr. SM Balaji, a superlatively dynamic “activist” in the dental and oral surgical profession, is the Secretary of the ISDR. The Scientific Chairman was Dr. S Shivakumar, yet another periodontist, who is seldom seen, much less heard, but nevertheless a melting pot of enterprise and energy when it comes to scientific sessions.

One thing that caught the eye was the layout of the scientific sessions in the form of debates rather than lengthy discourses. The theme was “Biomaterials Research” and there were two debates in Periodontics: “Biomaterials – Are they effective in Periodontics” and “Surgical vs non surgical periodontal treatment”. The teams “for” and “against” had two members each at the Reader level and one Team Leader at the Professor level. Each team bid their reviews against each other, within a time allotted of 8 minutes for each speaker and were judged by a panel of three Senior Professors, who would then wind up the session with their comments and possibly a consensus on the current opinion.

I cannot tell you what a welcome relief the structure was, being such a departure from the brouhaha that we have become accustomed to in our conferences today. The content which the younger faculty came up with was crisp, concise, clear, and cutting edge based on a diligent analysis of reviews on both topics. Really, it got me thinking that we have such a hidden wealth of tremendous resource in the younger faculty of today’s institutions that is so sparingly used in conferences and post graduate workshops today, which is almost a crime.

Also worth mentioning is The International Association of Dental Research (IADR), publishers of the most respected *Journal of Dental Research*, to which the ISDR is affiliated, is an organization started in 1970 but got its first international (read non-American) President only last year in Dr. Maria Fidela de Lima, from Brazil. It was truly uplifting to see the President of the IADR being a woman researcher from an emerging country and listening to her. She was so full of beans on how emerging countries like ours should dominate dental research in the future. She quoted Brazil, where it is now mandatory for at least one-third of the faculty to be full time and involved in research. This moved the ranking of Dentistry in Brazil from the 14^th^ to the 2^nd^ place in the choice of favored specialties in Medicine. I am sure, with the recent changes which the Dental Council of India has brought in with regard to publications, we too can hope to see a tremendous increase in the research potential in our institutions, provided we can encourage and bring our younger faculty to the forefront.

Hopefully, we should be taking a leaf out of this and putting up our younger members in the forefront in our efforts to take our profession forward. It is time we realized that the base of the pyramid is not only large but also tremendously gifted with talent and resource.

